# Molecular Identification of the “Facciuta Della Valnerina” Local Goat Population Reared in the Umbria Region, Italy

**DOI:** 10.3390/ani10040601

**Published:** 2020-04-01

**Authors:** Simone Ceccobelli, Emiliano Lasagna, Eymen Demir, Giacomo Rovelli, Emidio Albertini, Fabio Veronesi, Francesca Maria Sarti, Daniele Rosellini

**Affiliations:** 1 Department of Agricultural, Food and Environmental Sciences, University of Perugia, Borgo XX giugno 74, 06121, Italy; simone.ceccobelli@unipg.it (S.C.); eymendemir@akdeniz.edu.tr (E.D.); giacomo.rovelli@studenti.unipg.it (G.R.); emidio.albertini@unipg.it (E.A.); fabio.veronesi@unipg.it (F.V.); daniele.rosellini@unipg.it (D.R.); 2 Department of Animal Science, Faculty of Agriculture, Akdeniz University, Antalya, 07058, Turkey

**Keywords:** animal biodiversity, *Capra hircus*, genetic distinctiveness, microsatellite markers, molecular traceability, SSR

## Abstract

**Simple Summary:**

The Facciuta goat originated from Valnerina, a geographic area in central Italy, including the adjacent parts of four regions: Umbria, Marche, Lazio, and Abruzzo. The aim of this study was to assess how useful microsatellite molecular markers are for the genetic discrimination of the local goat, Facciuta della Valnerina, compared with the two cosmopolitan breeds, Saanen and Camosciata delle Alpi, reared in the same geographic area. The results revealed a very clear separation between the local population (Facciuta della Valnerina) and the two reference goat breeds (Saanen and Camosciata delle Alpi). Furthermore, reducing the number of markers from 16 to 12 still allowed us to distinguish the local population, indicating that microsatellite markers are an inexpensive method to discriminate local livestock breeds. This could be a fast and inexpensive genomic tool to trace goat products and distinguish their origin.

**Abstract:**

Italy holds important genetic resources of small ruminant breeds. By distinguishing goat breeds at the DNA level, certification of products from specific breeds can be valorized. The aim of this study was to establish the genetic identity of Facciuta della Valnerina, a local goat population of Italy, compared with the cosmopolitan breeds, Saanen and Camosciata delle Alpi, reared in the same geographic area. A total of 116 microsatellite alleles ranging from 4 to 13 were detected at 16 loci in the three goat populations/breeds. A total of 23 private alleles with frequencies lower than 0.3 were detected in the Facciuta della Valnerina population. The mean numbers of alleles were 6.67, 4.58, and 4.92 in Facciuta della Valnerina, Camosciata delle Alpi, and Saanen, respectively. The expected heterozygosity ranged from 0.20 to 0.86. Most loci were highly polymorphic and informative (polymorphic information content ≥0.50). Factorial correspondence analysis and principal components analysis revealed very clear separation between Facciuta della Valnerina and the two reference goat breeds. Reducing the number of markers from 16 to 12 (on the basis of polymorphic information content and the number of alleles) still allowed us to distinguish the local population, indicating that microsatellite markers are capable of discriminating local livestock breeds at a low cost.

## 1. Introduction

Goat (*Capra hircus*) is one of the most widespread livestock species in the world, comprising about 218 million goat heads in 2017. Asia has the largest proportion of the world population (52%), followed by Africa (39%), Europe (5%), the Americas (4%), and Oceania (<1%) [1]. Compared to other species (i.e., cattle), goats show a higher adaptability to different climatic and environmental conditions, a milder character, and a better ability to use forages [2]. Goats provide valuable milk and meat products [3], and goat meat prices are lower compared to other ruminant species. In terms of nutritional value, goat meat is appreciated for low fat (both in terms of intramuscular fat and fat deposits) and high protein content [4]. Moreover, it is characterized by a marked and unique flavor, which makes goat meat suitable for a variety of gastronomic preparations [5].

The preservation of local breeds is necessary to limit the loss of genetic resources, in particular for the species that are more important for food production, rural development, and environmental protection [3]. Among the actions aimed at preserving biodiversity, promotion, and valorization of local breeds, food products can be particularly effective [6]. The association between product and breed might be a way to satisfy consumer demand for specialty products, which, in turn, may improve the economic sustainability of local breeds [7]. Italy has a large variety of local breeds and typical products derived from them. Many of these typical products have obtained EU Protected Designation of Origin (DOP) or Protected Geographical Indication (IGP) labels, and many others are recognized by trademarks [3] to preserve their uniqueness.

Following the EU regulation 1825/2000, a mandatory labeling system for beef, sheep, and goat products was implemented to protect public health and to guarantee food safety [8]. Accordingly, each cut of meat must show a label carrying an alphanumeric identification called a "batch number" that identifies an animal, or a group of animals, and the country where the animal was born, reared, slaughtered, and sectioned. However, as pointed out by several authors, this system does not fully prevent frauds and errors along the production chain [9,10]. Animal identification using DNA-based techniques could address this problem, since DNA is unalterable throughout animal life and is present in derived products [11,12]. DNA-based identification could be extremely useful for traceability. However, the cost of using DNA analysis is one of its major limitations, and research has been carried out to develop fast and low-cost tests by using a low number of DNA markers [13,14,15,16,17]. Microsatellite markers or simple tandem repeats (STR), available for all livestock species, are commonly used for many applications such as parentage analysis and breed assignment [18]. These molecular markers are highly polymorphic, codominant, easily scored, and therefore very suitable to study small populations [19,20].

The aims of the present study were to establish the genetic differences and to indicate which alleles and which loci best describe the differences between the local population “Facciuta della Valnerina” (FAC) goat and two cosmopolitan breeds, Saanen (SAA) and Camosciata delle Alpi (CAM), that are widespread in the same geographic area, with the ultimate aim of valorizing the local population and exploiting its products.

## 2. Materials and Methods

### 2.1. Animal Sampling

A total of 24 blood samples of FAC were collected from three randomly taken animals (both sexes) per each of eight different flocks, all reared in Valnerina and Perugia, Italy. The approximate estimate of the current census of this population is around 200 heads, distributed in the areas mentioned and reared together with other goat breeds. Photos and supplementary information about the population studied are furnished in Table S1. The Vacutainer system was employed, using tubes containing an EDTA solution as an anticoagulant. The samples were transported at room temperature to the lab and then stored at −20 °C until analyses were performed. The analyzed animals can be considered as a representative sample of the population of FAC goats, since they were chosen trying to avoid closely related individuals in different farms that never exchanged bucks. In addition, DNA samples of 10 SAA and 10 CAM individuals (provided by the Italian Goat Consortium; http://www.goatit.eu/) were included as out-groups representative of cosmopolitan breeds reared in Italy. No ethical approval was required, in compliance with the European Directive 2010/63/UE and the Italian Regulation D. Lgs n. 26/2014, because samples were taken during obligatory routine animal sanitary controls by an authorized veterinarian.

### 2.2. Molecular Analyses

The GenElute Blood Genomic DNA kit (Sigma Aldrich, St. Louis, MO, USA) was used to extract the genomic DNA. Sixteen microsatellite loci (Table 1) were selected according to the recommendations of FAO and the International Society for Animal Genetics (ISAG) for genotyping and parentage analyses in goat breeds [21]. The markers were selected based on their degree of polymorphism and their position in the goat genome. STR markers were grouped in multiplex PCR according to reaction conditions and expected fragment sizes as reported by [22]. PCR products were separated by electrophoresis, with an automatic sequencer (ABI PRISM 3130xl, Applied Biosystems, Foster City, CA) according to the manufacturer’s recommendations. Allele sizes were estimated by using the internal size standard GeneScan-400 HD ROX (Applied Biosystems, Foster City, CA). Genotypes were visualized and interpreted with GeneMapper software, version 5.0 (Applied Biosystems, Foster City, CA).

### 2.3. Statistical Analysis

Allele frequencies, mean number of alleles, polymorphic information content (PIC) for each STR locus, and the observed and expected heterozygosity in the three populations/breeds were calculated using the Microsatellite Toolkit software [23]. The HP-RARE version 1.0 software was used to calculate average allelic richness for each population/breed (Rt), allowing comparisons among different sample sizes [24]. A test for departure from the Hardy–Weinberg equilibrium (HWE) was performed using a Markov chain Monte Carlo method (20 batches, 5000 iterations per batch, and a dememorization number of 10,000) implemented in the GENEPOP version 4.0 software [25]. The levels of significance were adjusted using the false discovery rate (FDR) procedure [26]. Population subdivision was investigated by calculating the global multilocus *F*_ST_ value. The pairwise *F*_ST_ index between populations [27] was estimated using the Arlequin 3.5 software [28], and their associated 95% confidence intervals (IC_95%_) were calculated using the GDA software [29]. Factorial correspondence analysis (FCA) [30], carried out with GENETIX 4.05, was used to further investigate the differentiation of the breeds. To investigate the distinctiveness of each breed when adopting an approach without assumptions about HWE or linkage disequilibrium, discriminant analysis of principal components (DAPC) was carried out with the method implemented in the ADEGENET software package [31] within the statistical package R version 3.6.2 [32]. A multivariate DAPC analysis performs a preliminary data transformation step using principal component analysis (PCA) to create uncorrelated variables that summarize total variability (e.g., within and between groups). These variables are then used as input to discriminant analysis (DA), which aims to maximize between-group variability and achieve the best discrimination of individuals into predefined clusters. DAPC was conducted without a posteriori group assignments by inferring the most likely number of genetic clusters (*K*) using the *find.clusters* function of ADEGENET. This function utilizes *K*-means clustering to calculate a Bayesian information criterion (BIC) value for each potential value of *K* (the most likely *K* has the lowest BIC value) and delineates individual group assignments for DAPC.

## 3. Results and Discussion

### 3.1. Genetic Variation

The number of observed alleles (Na), together with the expected heterozygosity (H_E_) and observed heterozygosity (H_O_), PIC values, and Hardy–Weinberg equilibrium test for each locus are presented in Table 1. A total of 116 alleles were found for the sixteen microsatellites analyzed, ranging from 4 (ETH10 and MAF209) to 13 (HSC) alleles per locus. The mean number of alleles per locus over all breeds was 7.25. The expected heterozygosity varied from 0.86 at HSC to 0.20 at MAF209, and the average across all loci was 0.65, indicating a moderate genetic diversity across the three goat breeds. The mean PIC ranged from 0.18 to 0.80, with a mean value of 0.60. Due to its low PIC value, also observed in other Italian and foreign breeds [33,34,35,36], the MAF209 marker was excluded for further statistical analysis. The remaining 15 loci had PIC ≥ 0.50 and therefore were highly informative. Since significant deviation from the Hardy–Weinberg equilibrium was detected for the loci OarFCB11, CRSM60, and ILST19, they were excluded from further statistical analysis. The mean number of alleles per locus ranged from 4.58 for CAM to 6.67 for FAC (Table 2). After adopting the rarefaction methodology [24], the mean allelic richness ranged from 4.36 (CAM) to 5.17 (FAC) in a sample size of eight individuals. Lower allelic diversity was found in many local goat breeds [37,38,39,40], but higher MNA values were reported in both Italian [41,42,43] and foreign [20,44] breeds. FAC had higher observed heterozygosity compared to the cosmopolitan breeds, with H_O_ of 0.68. Although lower than H_E_ (0.74), this value of H_O_ is similar to that reported for other Italian or foreign breeds [40,41,44]. Higher H_E_ values were reported in other cases [20,37,38]. The presence of private alleles (i.e., alleles present in one breed and absent in the others) were observed in all three populations/breeds, but were about 5-fold more abundant in FAC (25 in FAC, 4 in CAM and 5 in SAA). Considering the allele distribution within the three breeds, it is possible to note the presence, both in CAM and SAA, of four alleles that are missing in FAC (Table 3); these differences can be used to trace monobreed products. The frequencies of the 25 private alleles of FAC ranged from 0.0217 to 0.7708. A similar number of private alleles (21) were reported in Sukuma goats [40], while lower numbers were reported in some Italian goat breeds such as Alpine and Girgentana [41,43]. Again, this number is affected by the factors mentioned above.

### 3.2. Genetic Differentiation

**Figure 1:**
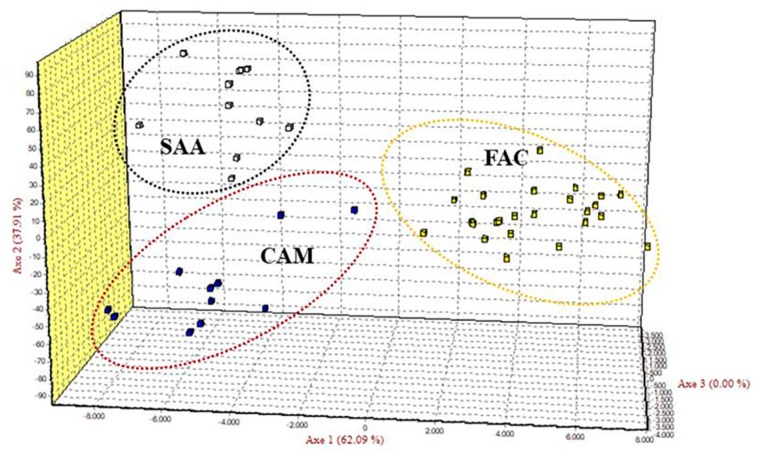
Factorial correspondence analysis of the three goat populations/breeds studied with 12 markers. FAC, Facciuta della Valnerina; CAM, Camosciata delle Alpi; SAA, Saanen.

**Figure 2:**
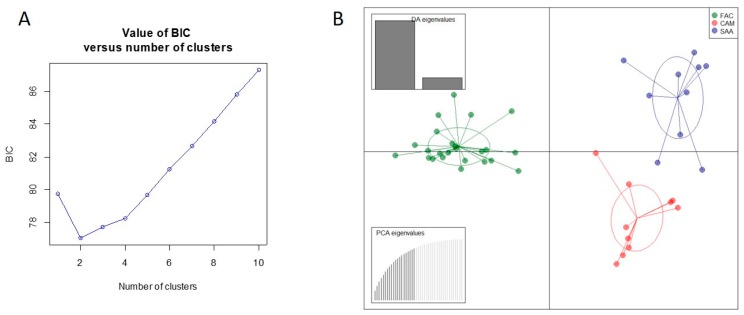
Results of discriminant analysis of principal components (DAPC). (**A**) Bayesian information criterion (BIC) values plotted for the number of clusters ranging from *K* = 1 to 10. (**B**) Scatterplot of the first two principal components of DAPC using populations as an a posteriori cluster. The individuals are assigned to populations a posteriori, that is, after automated determination of the number of clusters, instead of forcing them into known populations. Populations are labeled inside their 95% inertia ellipses, and dots represent individuals. The inset above indicates the eigenvalues of the first two principal components. The inset below represents the total variance explained by the principal components. FAC, Facciuta della Valnerina; CAM, Camosciata delle Alpi; SAA, Saanen.

Pairwise genetic differentiation indexes (*F*_ST_) were found significant (*p* < 0.001) for all the breeds (Table 4). In this study, the lowest (0.0729, IC_95%_ 0.042–0.141) and the highest (0.0928, IC_95%_ 0.060–0.109) pairwise *F*_ST_ values were detected between SAA and CAM and between FAC and SAA, respectively, with a mean of 0.084 (IC_95%_ 0.061–0.113). Additionally, the *F*_ST_ value between FAC and CAM was high (0.0897, IC_95%_ 0.038–0.131), indicating a clear-cut genetic differentiation between FAC and the cosmopolitan breeds. A previous study [21] reported similar mean *F*_ST_ value (0.085) in Small East African goats, while a lower mean *F*_ST_ value (0.07) was reported in eight Italian goat breeds [42]. The results of correspondence analysis further highlighted the genetic differentiation between the breeds (Figure 1) and sharply distinguished FAC individuals from those of the other breeds. A clear-cut differentiation between local goat breeds was shown by FCA analyses in other studies [20,42]. In the DAPC analysis, 25 principal components were retained as input for discriminant analysis, accounting for 84.5% of the total genetic variability. The Bayesian information criterion (BIC) statistic generated by discriminant analysis of principal components (DAPC) indicates that the optimal number of clusters in the data set is *K* = 2 (Figure 2A). On the scatterplot of the first two components of the DA (Figure 2B), FAC appeared distinct from both SAA and CAM. Hence, these results reinforce the evidence from the pairwise *F*_ST_ values and the factorial correspondence analysis, as observed in other studies [45,46].

## 4. Conclusions

The present study represents a first attempt to show the genetic distinctiveness of the local goat population of Facciuta della Valnerina in comparison to two cosmopolitan goat breeds (Saanen and Camosciata delle Alpi) using as little as 12 microsatellite markers. Four private alleles were detected for this local population, which can be used to trace monobreed products. Although the scope of this work was limited in terms of the number of populations/breeds and sample size, the results are sufficiently clear-cut to propose that these markers could be used for product traceability and market protection of products derived from Facciuta della Valnerina. The same methodology could be applied to other local goat breeds, with the objective of providing a molecular tool that could help to protect and valorize local genetic diversity in goats.

## Figures and Tables

**Table 1:** Characteristics of the SSR markers used for this study, relative to all 44 heads: chromosome position (Chr), size range (S.R.), number of alleles (Na), expected heterozygosity (H_E_) and observed heterozygosity (H_O_), mean polymorphic information content (PIC), number of breeds deviating from the Hardy–Weinberg equilibrium (HWE Breed). The markers excluded from further analysis on the bases of PIC values and/or deviation from HWE are shown in grey.

Locus	Chr.	S.R. (bp)	Na	H_E_	H_O_	PIC	HWE Breed †
INRA005	10	176–190	5	0.59	0.54	0.51	0
BM8125	17	110–130	9	0.71	0.63	0.63	1
CSRD247	14	220–247	8	0.65	0.57	0.59	1
HAUT27	26	128–158	7	0.77	0.82	0.71	0
TGLA122	21	137–181	8	0.75	0.78	0.68	0
HSC	20	267–301	13	0.86	0.78	0.80	0
MCM527	5	165–187	7	0.65	0.72	0.60	0
SRCRSP8	Not reported	215–255	9	0.52	0.56	0.50	0
BM1329	6	155–200	6	0.66	0.50	0.58	1
OarFCB11	2	122–140	7	0.75	0.71	0.70	2
MAF209	17	100–104	4	0.20	0.19	0.18	2
MAF65	15	116–158	10	0.75	0.52	0.68	1
CRSM60	Not reported	75–91	6	0.72	0.43	0.66	3
ETH10	5	212–224	4	0.46	0.44	0.50	0
ILSTS019	Not reported	142–162	6	0.78	0.78	0.72	2
SRCRSP5	21	156–178	7	0.64	0.76	0.57	0
**Total** (±SD)			116 ± 2.29	0.65 ± 0.16	0.61 ± 0.17	0.60 ± 0.15	

†: After Benjamini and Hochberg (1995) correction.

**Table 2:** Sample size of each population/breed (N), mean number of alleles (MNA), allelic richness per population/breed (Rt), number of private alleles (PA), and mean observed (H_O_) and expected heterozygosity (H_E_).

Population/Breed	N	MNA ± SD	Rt ^(1)^	PA	H_O_ ± SD	H_E_ ± SD
FAC	24	6.67 ± 2.10	5.17	25	0.68 ± 0.03	0.74 ± 0.03
CAM	10	4.58 ± 1.62	4.36	4	0.59 ± 0.05	0.63 ± 0.06
SAA	10	4.92 ± 1.38	4.56	5	0.64 ± 0.04	0.64 ± 0.04

^(1)^ Based on eight individuals. FAC, Facciuta della Valnerina; CAM, Camosciata delle Alpi; SAA, Saanen.

**Table 3:** Private alleles (frequencies in brackets) found in the three goat populations/breeds. Alleles in bold are present in CAM and SAA and absent in FAC.

Locus	Population/Breed		
	FAC	CAM	SAA
INRA5		113 (0.1000)	
BM8125	109 (0.0217)	123 (0.0500)	119 (0.0500)
	121 (0.0217)		
	127 (0.0217)		
CSRD247	216 (0.1304)	228 (0.3125)	228 (0.1111)
	232 (0.2174)	234 (0.1875)	242 (0.1250)
HAUT27		145 (0.0500)	145 (0.1000)
TGLA122	147 (0.0455)	133 (0.1000)	
HSC	268 (0.0217)		266 (0.0500)
	276 (0.0435)	270 (0.0500)	270 (0.2000)
	278 (0.0435)		
	296 (0.0217)		
MCM527	160 (0.1304)		
SRCRSP8	218 (0.0217)		224 (0.0500)
	230 (0.0217)		242 (0.1000)
	238 (0.0435)		
BM1329	174 (0.1087)		
	180 (0.0870)		
MAF65	117 (0.0870)		
	119 (0.0435)		
	125 (0.1957)		
	127 (0.0435)		
	129 (0.2391)		
MAF209	105 (0.7708)	101 (0.0500)	101 (0.0500)
	107 (0.1042)		
SRCRSP5	161 (0.1250)		
	179 (0.0313)		

FAC, Facciuta della Valnerina; CAM, Camosciata delle Alpi; SAA, Saanen.

**Table 4:** Pairwise and global *F*_ST_ distance (with confidence intervals at 95%—IC_95%_) between the three goat populations/breeds studied with 12 markers.

Population/breed	N	FAC	CAM	SAA
**FAC**	24	0.0000		
**CAM**	10	0.0897 (0.038–0.131)	0.0000	
**SAA**	10	0.0928 (0.060–0.109)	0.0729 (0.042–0.141)	0.0000
	Global *F*_ST =_ 0.084 (0.061–0.113)			

FAC, Facciuta della Valnerina; CAM, Camosciata delle Alpi; SAA, Saanen; N, sample size of each population/breed.

## Supplementary Materials

The following are available online at https://www.mdpi.com/2076-2615/10/4/601/s1 Table S1: Description of the “Facciuta della Valnerina” population.
