# Supplementary material for: Molecular Identification of the “Facciuta Della Valnerina” Local Goat Population Reared in the Umbria Region, Italy
Source: Animals (Basel). 2020 Apr 1;10(4):601. doi: 10.3390/ani10040601 (PMC7222817; doi:10.3390/ani10040601)
Supplement: Supplementary file 1 [file animals-10-00601-s001.pdf]

Table S1. Description of the Facciuta della Valnerina population.

The Facciuta goat originated from an area that includes Valnerina (Southern Umbria), the Vissano and Camerinese areas (Marche region), Sabina area (Lazio region) and some zones of Northern Abruzzo region. The Valnerina goat is also known with names that refer to its most striking feature, which are two white lists along the face, sometimes take the name of "Rigatina", "Facciuta Bianca" and "dalla Mascherina". The hairs are long and the coat colour is tan black with reddish reflections, while the extremities of the limbs are white or light beige like the belly and the perianal area; some animals, however, can show a clear coat along the flank. Some subjects have a coat completely red with extremities, belly and perianal area white. The size is medium-large, with a large, triangular, elongated and well-proportioned head. The face profile can be straight or slightly hilly and on the head there's the distinctive trait: two light stripes (frisature) along the face and at eye level. The ears are medium-large and semi-upright.

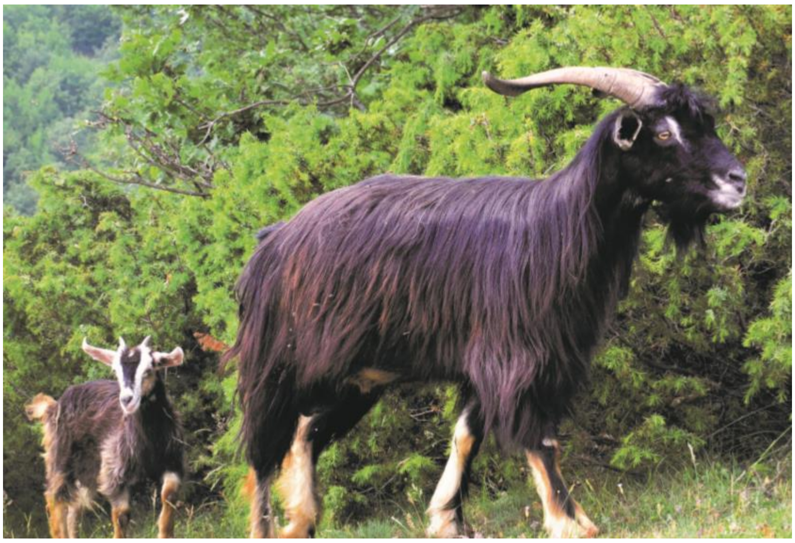

The beard is present in both sexes as well as a tuft of shaggy hair in the frontal area; both the goats and bucks are also endowed with flat divergent horns. In the females the horns can reach 30 cm in length and assume the lyre-like shape, while in the males the horns can reach 50 cm. Animals without horns are also frequent. The length of the trunk is considerable, the thorax and abdomen are large and the dorsal line is straight. The limbs are long and robust and well adapted to rough bushed and wooded environments.
These animals are rather early, the average age at first delivery is around 12 months and the fertility is high, so trigeminal offsprings are very frequents.
The Valnerina goat is bred with traditional grazing systems, with short periods of winter housing. The production of the kid is very interesting, due to the size reached in a short time; milk production is not negligible, with the advantage, for the not dairy production farms, that the lactation can be strongly reduced, within few days after separation from the kid.
The approximate estimate of the current census of this breed is around 200 heads, distributed in the mentioned areas and reared together with other goats.

References

1. I Quaderni della Biodiversità, Numero 7, Capra Facciuta della Valnerina, racconto di una popolazione caprina dimenticata. Available online: http://biodiversita.umbria.parco3a.org/pagine/pubblicazioni-000 (accessed on 08 January 2020).
2. Italian Goat Consortium. Available online: http://www.goatit.eu/ (accessed on 08 January 2020).
